# Pan-cancer analysis identifies PD-L2 as a tumor promotor in the tumor microenvironment

**DOI:** 10.3389/fimmu.2023.1093716

**Published:** 2023-03-16

**Authors:** Jingfang Lv, Zheng Jiang, Junhu Yuan, Meng Zhuang, Xu Guan, Hengchang Liu, Yefeng Yin, Yiming Ma, Zheng Liu, Hongying Wang, Xishan Wang

**Affiliations:** ^1^ Department of Colorectal Surgery, National Cancer Center/National Clinical Research Center for Cancer/Cancer Hospital, Chinese Academy of Medical Sciences and Peking Union Medical College, Beijing, China; ^2^ State Key Laboratory of Molecular Oncology, National Cancer Center/Cancer Hospital, Chinese Academy of Medical Sciences and Peking Union Medical College, Beijing, China

**Keywords:** immune checkpoint, PD-L2, tumor associated macrophages, colon cancer, pan-cancer analysis

## Abstract

**Background:**

Programmed cell death protein 1 (PD-1) receptor has two ligands,programmed death-ligand 1 (PD-L1) and PD-L2. When compared with PD-L1, PD-L2 has not received much attention, and its role remains unclear.

**Methods:**

The expression profiles of *pdcd1lg2* (PD-L2-encoding gene) mRNA and PD-L2 protein were analyzed using TCGA, ICGC, and HPA databases. Kaplan-Meier and Cox regression analyses were used to assess the prognostic significance of PD-L2. We used GSEA, Spearman’s correlation analysis and PPI network to explore the biological functions of PD-L2. PD-L2-associated immune cell infiltration was evaluated using the ESTIMATE algorithm and TIMER 2.0. The expressions of PD-L2 in tumor-associated macrophages (TAMs) in human colon cancer samples, and in mice in an immunocompetent syngeneic setting were verified using scRNA-seq datasets, multiplex immunofluorescence staining, and flow cytometry. After fluorescence-activated cell sorting, flow cytometry and qRT-PCR and transwell and colony formation assays were used to evaluate the phenotype and functions of PD-L2^+^TAMs. Immune checkpoint inhibitors (ICIs) therapy prediction analysis was performed using TIDE and TISMO. Last, a series of targeted small-molecule drugs with promising therapeutic effects were predicted using the GSCA platform.

**Results:**

PD-L2 was expressed in all the common human cancer types and deteriorated outcomes in multiple cancers. PPI network and Spearman’s correlation analysis revealed that PD-L2 was closely associated with many immune molecules. Moreover, both GSEA results of KEGG pathways and GSEA results for Reactome analysis indicated that PD-L2 expression played an important role in cancer immune response. Further analysis showed that *PD-L2* expression was strongly associated with the infiltration of immune cells in tumor tissue in almost all cancer types, among which macrophages were the most positively associated with PD-L2 in colon cancer. According to the results mentioned above, we verified the expression of PD-L2 in TAMs in colon cancer and found that PD-L2^+^TAMs population was not static. Additionally, PD-L2^+^TAMs exhibited protumor M2 phenotype and increased the migration, invasion, and proliferative capacity of colon cancer cells. Furthermore, PD-L2 had a substantial predictive value for ICIs therapy cohorts.

**Conclusion:**

PD-L2 in the TME, especially expressed on TAMs, could be applied as a potential therapeutic target.

## 1 Introduction

T cell-based immune systems have evolved to recognize and destroy aberrant cells, such as pathogen-infected and cancer cells. According to the model for T cell activation proposed by Kevin Lafferty et al. (1), T cells require two signals to become fully activated. The first signal is provided by the binding of the T cell receptor on T cells to peptide-major histocompatibility complexes on target cells. The second signal, which is delivered to T cells by antigen-presenting cells to promote T cell clonal expansion, cytokine secretion, and effector functions, is an antigen-independent co-stimulatory signal. The discovery of the B7:CD28 family has revealed co-stimulatory pathways that can provide positive and negative second signals to antigen-experienced effector T cells and regulate the quantity and functional activity of antigen-specific T cells (2). Programmed cell death protein 1 (PD-1) receptor and its ligands, programmed death-ligand 1 (PD-L1) and PD-L2, are the most notable pathways in the B7:CD28 family. PD-L1 encoding gene *CD274* and PD-L2 encoding gene *pdcd1lg2* are located adjacent to each other on chromosome 9p24.1, and there is a 23-kb non-coding region in mouse and 42-kb in human between these two genes (3). The amino acid sequence homology between PD-L1 and PD-L2 is approximately 40 percent (4). Currently, many immune therapies that target the PD-1 axis include monoclonal antibodies against PD-1 and PD-L1. Despite the considerable improvement in patient outcomes has been achieved with anti-PD-1/PD-L1 targeted therapies, durable responses to these therapies are observed in only few patients and intrinsic therapy resistance is common (5, 6). Therefore, it is crucial to discover a new therapeutic target and identify the biomarkers for immunotherapy. Compared with PD-L1, PD-L2 has received far less research attention and its role in modulating tumor progression remains unclear. Several studies described a T cell inhibitory function for PD-L2. PD-1:PD-L2 interaction resulted in inhibition of proliferation and cytokines production of T cells (7). Katharina Pfistershammer and colleagues also revealed that PD-L2 inhibited T cell activation and cytokines production in primary as well as in pre-stimulated T cells (8). In line with these results, blocking of PD-L2 on dendritic cells (DCs) (9) and endothelial cells (10) enhanced their T cell stimulatory capacity. Additionally, it had been found that PD-L2 was also involved in intracellular signaling pathways to promote tumor cell migration, invasion, and induce drug resistance indicating that PD-L2 expression on tumor cells was also involved in evading antitumor immunity (11, 12). By contrary, Liu X et al. found that the expression of PD-L2 on murine tumor cells could promote CD8^+^T cell expansion and enhance CD8^+^T cell mediated rejection of tumor cells (13). Similarly, PD-L2 expressed by DCs stimulated T cell proliferation and induced a distinct pattern of lymphokine secretion (14). Evidences obtained from *in vitro* and *in vivo* experiments in PD-L2 conventional knockout mice also demonstrated that PD-L2 played a predominantly tuning molecule role in the generation of both T helper 1 and cytotoxic T lymphocyte responses (15). Intriguingly, studies on PD-1-deficient mice showed that PD-L2 could still interact with and convey costimulatory effects to PD-1^-/-^ T cells, raising the hypothesis of a second, costimulatory receptor in tumor microenvironment (TME) (13). Collectively, it is currently unclear what are the roles of PD-L2 in modulating tumor progression.

Here, we conducted a pan-cancer analysis for the first time and performed *in vitro* and *in vivo* studies to illustrate the prevalence, prognostic and predictive values, and biological functions of PD-L2 in cancers to find out which aspects future studies should focus on.

## 2 Materials and methods

**Figure 1:**
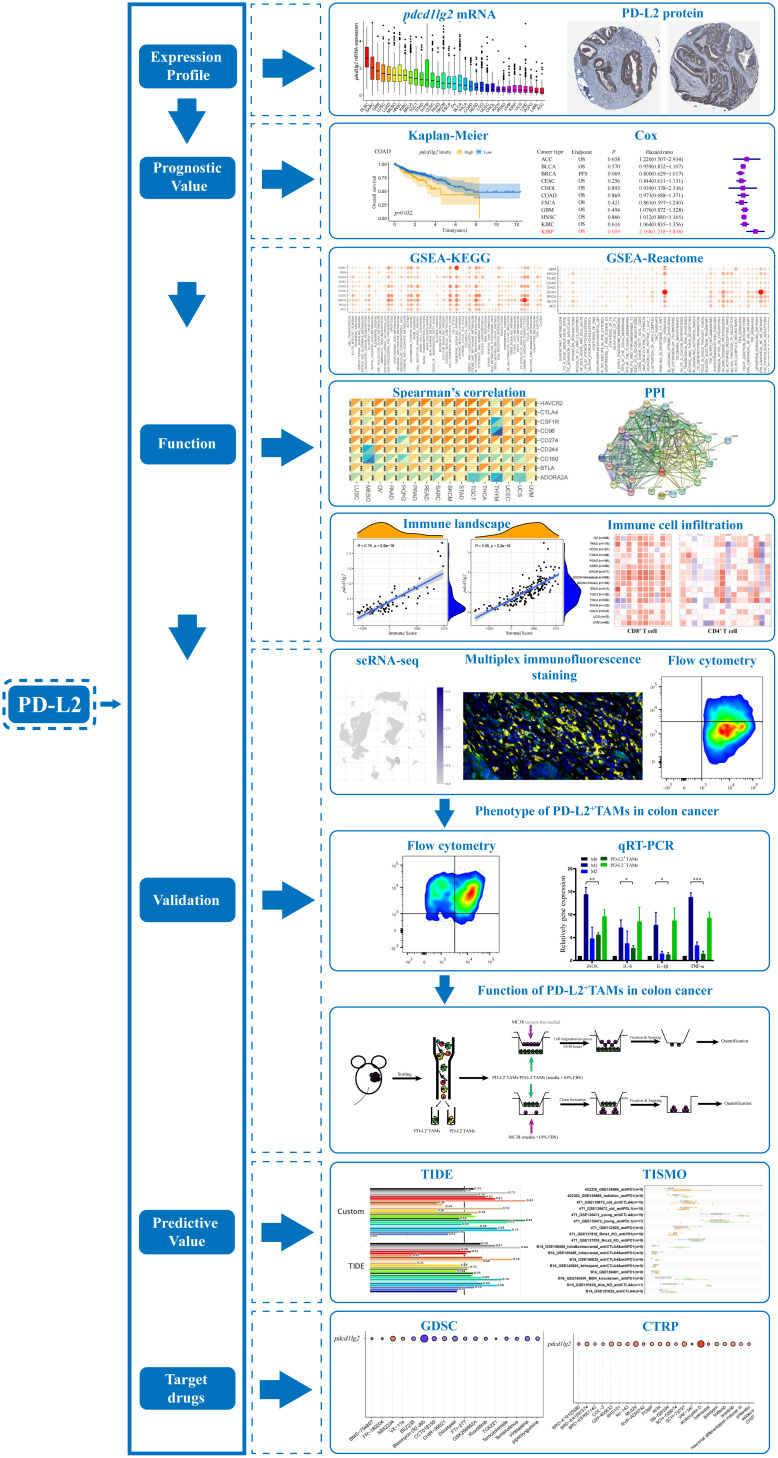
The flow chart of the entire study. PD-L2, programmed cell death 1 ligand 2; GSEA, gene set enrichment analysis; KEGG, Kyoto Encyclopedia of genes and genomes; PPI, protein-protein interaction; scRNA-seq, single-cell RNA sequencing; TAMs, tumor associated macrophages; qRT-PCR, quantitative reverse transcription-polymerase chain reaction; TIDE, Tumor Immune Dysfunction and Exclusion; TISMO, Tumor Immune Syngeneic MOse; GDSC, Genomics of Drug Sensitivity in Cancer; CTRP, Cancer Therapeutics Response Portal.

The study flow chart is presented in **Figure 1**.

### 2.1 *Pdcd1lg2* mRNA expression profile analysis

The *pdcd1lg2* mRNA expression in various types of tumors was analyzed based on The Cancer Genome Atlas (TCGA) and International Cancer Genome Consortium (ICGC) databases. Since the data from both the databases are publicly available, the present study was exempted from the approval of local ethics committees. Abbreviations for cancer is presented in **Supplementary Table 1**.

Cancer tissue RNA sequencing data from TCGA pan-cancer data were downloaded from UCSC Xena (http://xenabrower.net/) (16). Normalized gene expression values were converted to transcripts per million (TPM) and log-transformed (log_10_((normalized_count*1e^6^)+1)). Normalized RNA sequencing data for all available BOCA-FR, BPLL-FR, BRCA-FR, LICA-FR, LIRI-JP, ORCA-IN, OV-AU, PACA-AU, PACA-CA, PRAD-CA, PRAD-FR, and RECA-EU samples were downloaded from the ICGC data portal (http://dcc.icgc.org/). Normalized gene expression values were log-transformed (log_10_(normalized_count+1)).

### 2.2 PD-L2 protein expression profile analysis

PD-L2 protein expression levels in 19 types of tumor tissues were verified using immunohistochemistry (IHC) from the Human Protein Atlas (HPA) database (http://www.proteinatlas.org/) (17).

### 2.3 Prognostic analysis

The *pdcd1lg2* expression profiles from TCGA pan-cancer data were used for prognostic analysis. Data on survival was obtained from the UCSC Xena database. Overall survival (OS) is the period from the date of diagnosis until the date of death from any cause. Progression-free survival (PFS) is the period from the date of diagnosis until the date of the first occurrence of a new tumor event, which includes progression of the disease, locoregional recurrence, distant metastasis, new primary tumor, or death with tumor. Bivariate *pdcd1lg2* expression levels with the cut-off chosen by the “surv-cutpoint” function of the “survminer” R package were used to perform Kaplan-Meier curve analysis to assess the prognostic role of *pdcd1lg2*. Moreover, *pdcd1lg2* continuous variable expression data were used in the Cox regression analysis, and we calculated the hazard ratios (HR) and 95% confidence intervals (CI).

### 2.4 Gene set enrichment analysis (GSEA)

After ranking *pdcd1lg2* mRNA expressions, the data of each cancer from TCGA pan-cancer database were separated into low- (bottom 30%) and high-*pdcd1lg2* subgroups (top 30%). GSEA was used to determine the potential biological and molecular functions of *pdcd1lg2* in each cancer and was carried out using GSEA software (18, 19). The gene expression datasets of low- and high-*pdcd1lg2* subgroups were submitted to GSEA software (v.4.2.3). A two-class analysis with 1000 permutations of phenotype and weighted metric was used. After being downloaded from the Molecular Signatures Database (MSigDB, http://www.gseamsigdb.org/gsea/msigdb), the “gmt” files of the Kyoto Encyclopedia of Genes and Genomes (KEGG) gene sets (c2.cp.kegg.v7.5.1.symbols.gmt) and Reactome gene sets (c2.cp.reactome.v7.5.1.symbols.gmt) were used to calculate the normalized enrichment score (NES) and false discovery rate (FDR) for each biological process. Ggplot2 R package was applied to visualize the results.

### 2.5 Spearman’s correlation analysis

Spearman’s correlation analysis was performed by using the R function “cor.test” to show the associations between the *pdcd1lg2* mRNA and immune-related gene expression which were obtained from TCGA pan-cancer data and *p*<0.05 was considered significant.

### 2.6 Protein-protein interaction (PPI) analysis

STRING is an online database used for retrieving interactions among genes/proteins (http://string-db.org/cgi/input.pl) (20). We performed PPI analysis using the STRING website with high-throughput experimental data, literature, and predictions based on genomic context analysis. A confidence score above 0.7 was set as the cut-off criterion.

### 2.7 Immune cell infiltration analysis

The ESTIMATE algorithm (21) by using the R package “estimate” was used to assess the correlation between *pdcd1lg2* expression and stromal cell infiltration (stromal score) and immune cell infiltration (immune score) in the tumor tissues from the TCGA dataset.

Tumor IMmune Estimation Resource (TIMER) is a data resource for analyzing immune cell infiltration across distinct cancers using various algorithms (22). The *pdcd1lg2*-associated immune cell infiltration correlations of the TCGA pan-cancer project were downloaded from the TIMER 2.0 database (http://timer.cistrome.org/) in the “Gene” function of the “Immune Association” section. We visualized the statistical Spearman’s correlations between *pdcd1lg2* mRNA expression and 21 immune cell subsets, including CD8^+^T cell, CD4^+^T cell, regulatory T cell (Treg), B cell, neutrophil, monocyte, macrophage, dendritic cell (DC), natural killer (NK) cell, mast cell, cancer-associated fibroblast (CAF), progenitor of lymphoid cell, progenitor of myeloid cell, progenitor of granulocyte-monocyte, endothelial cell (Endo), eosinophil (Eos), hematopoietic stem cell (HSC), T cell follicular helper (Tfh), γ/δ T cell, NK T cell (NKT), and myeloid-derived suppressor cell (MDSC) across cancers in a heatmap.

### 2.8 Single-cell RNA sequencing analysis

Tumor Immune Single-cell Hub (TISCH, http://tisch.comp-genomics.org/home/), including 79 high-quality single-cell datasets, is used to screen for scRNA-seq datasets with detailed cell-type annotation at the single-cell level focusing on tumor microenvironment across different cancers (23). Based on MAESTRO v.1.1.0 (24), all the collected datasets are uniformly processed with a standardized workflow, including quality control, batch effect removal, cell clustering, differential expression analysis, cell-type annotation, malignant cell classification and GSEA. Briefly, low quality cells are filtered out if the number of total counts per cell is <1000, or the number of detected genes per cell is <500. The entropy-based metric (25, 26) is employed to quantify the mixing of the data across batches. The datasets with a median entropy lower than 0.7 are corrected the batch effect using Seurat v.3.1.2 (27). The MAESTRO workflow identifies the top 2000 variable features and employs principal component analysis for dimension reduction, K nearest neighbors, and Louvain algorithm for identifying clusters for each dataset (28, 29) and the uniform manifold approximation and projection is used to reduce the dimension further and visualize the results of clusters (30). For each cluster, TISCH utilizes the Wilcoxon test to identify differentially expressed genes (DEGs) based on the log-transformed fold change (|logFC|>=0.25) and FDR (FDR<10^-6^) and annotates the cell clusters with a marker-based annotation method employed in MAESTRO based on the DEGs. The marker genes of each cell type are collected from the published resources (31–33). Moreover, TISCH also performs manual corrections to all the annotated cell types by combining them with original annotation and malignant cell identification. There are three sources that TISCH combines to identify the clusters of malignant cells, cell-type annotations provided by the original studies, the expression of malignant cell markers from initial research, and the prediction of InferCNV v.1.2.1 (34) based on the predicted copy number variation. After the streamlined processing, TISCH curates the cell-type annotation of all datasets at three levels: malignancy, major-lineage and minor-lineage. In this study, we enrolled GSE166555 and EMTAB8107 to analyze the *pdcd1lg2* expression distribution.

### 2.9 Multiplex immunofluorescence staining

The 5 μm sections of the formalin-fixed, paraffin-embedded colon cancer tissue specimens were obtained from patients hospitalized in the department of colorectal surgery of the National Cancer Center after surgery. Informed consent was obtained from all patients enrolled in this study. The medical ethics committee of the National Cancer Center permitted the use of tissues obtained from clinical excision. The included patients, diagnosed with colon adenocarcinoma using histopathological evaluation, did not have a history of autoimmune disease and neoadjuvant chemotherapy or radiotherapy before surgical resection.

Multiplex staining and multispectral imaging to identify the co-expression of PD-L2 and macrophage marker CD68 in tumor microenvironment (TME) was performed as previously described (35). Briefly, the slides were deparaffinized in xylene and rehydrated in ethanol. Antigen retrieval was carried out in citrate buffer (PH 6.0) using microwave heating. The primary antibodies, PD-L2 (Rabbit, 1:100, CST, Danvers, Massachusetts, US) and CD68 (Rabbit, 1:800, Danvers, Massachusetts, US), were sequentially incubated for 1 h in a humidified chamber at room temperature. Detections using the rabbit SuperPicture Polymer Detection HRP kit (Life Technologies, CA), visualizations of each target using fluorescently labeled Tyramide signal amplification (TSA) (1:50, Life technologies, Grand Island, NY), immersing the slide in citrate buffer (PH 6.0) and heating using microwave heating were performed after each incubation of primary antibody followed by incubation with horseradish peroxidase-conjugated secondary antibody incubation and tyramide signal amplification. Nuclei were stained with 4’-6’-diamidino-2-phenylindole (DAPI) (Sigma-Aldich, Saint Louis, Missouri, US). Multispectral images were analyzed, and positive cells were quantified at a single-cell level using the inForm image analysis software (version 2.4, PerkinElmer, Waltham, Massachusetts, US).

### 2.10 Cell line, mice and animal model

The MC38 mouse colon cancer cell line was obtained from Procell Life Science and Technology Co., Ltd. (Wuhan, China). Cells were grown in RPMI 1640 medium supplemented with 10% fetal bovine serum (FBS), and penicillin-streptomycin mix (all from Gibco/Invitrogen Technologies, Waltham, Massachusetts, US) at 37°C in a humidified incubator with 5% CO2.

Around 6-8-week-old (18-22g) C57/B6J male mice were housed in the animal care unit of the National Cancer Center. All animal experimental protocols were approved by the ethics committee of the Chinese Academy of Medical Sciences, National Cancer Center. MC38 cells (5 × 10^5^) in 200 μL were subcutaneously injected into the flank of each mouse. Tumors were measured two times per week by caliper, and tumor volumes were calculated using the modified ellipsoid formula 1/2 × (length × width^2^). At 7 d, 14 d, 21 d, and 28 d after cell injection, the mice were sacrificed to collect the tumors, which were examined using flow cytometry or fluorescence-activated cell sorting (FACS).

### 2.11 Flow cytometry

In accordance with the manufacturer’s instructions, the tumors were chopped and then digested using enzymes from the tumor dissociation kit (Miltenyi Biotec, Cologne, Germany) at 37°C for 41 min to obtain single-cell suspensions. Viable cells were counted after filtering the digested samples through 70-μm Falcon cell strainers. Loosely attached cells were collected by washing the strainer with 5 mL phosphate-buffered saline (PBS). The cells were collected by centrifuging the cell suspension for 8 min at 300 ×g. Then, the samples were processed into single-cell suspensions and blocked with TruStain FcX (BioLegend, San Diego, California, US). Tumor associated macrophages (TAMs) were incubated with antibodies: FITC anti-mouse CD45 (clone: I3/2.3), BV421 anti-mouse CD11b (clone: M1/70), PE/Cy7 anti-mouse F4/80 (clone: BM8), APC anti-mouse PD-L2 (clone: TY25), and PE anti-mouse CD206 (clone: C068C2) (all from BioLegend, San Diego, California, US), for 15 min in the dark at 4°C. The cells were then washed twice with 4 mL flow buffer, centrifuged (300 ×g, 5 min), and re-suspended in 500 μL flow buffer for analysis. For intracellular staining, the surface antigens-labeled cells were fixed and permeabilized with 4% paraformaldehyde/1% Triton X-100, and subsequent staining was performed following specific antibody protocols. Flow cytometry was carried out using a FACSCalibur flow cytometer (BD Biosciences, Franklin Lakes, New Jersey, US). Flow cytometry data analysis was performed using the FlowJo software (FlowJo, US).

### 2.12 FACS

Five tumors per group were isolated and digested as described above. Then, 2 × 10^7^ cells in PBS were stained with LIVE/DEAD Fixable Blue for 30 min at room temperature in the dark. After washing the cells with FACS buffer (sterile PBS with 3% BSA), TrueStain FcX in FACS buffer was used to block cells for 20 min at room temperature. Cells were subsequently incubated with FITC anti-mouse CD45, BV421 anti-mouse CD11b, PE/Cy7 anti-mouse F4/80, and APC anti-mouse PD-L2 as mentioned above in the dark at 4°C for 20 min, re-suspended in 500 µL FACS buffer, and sorted using the BD Biosciences FACSAria III. CD45^+^CD11b^+^F4/80^+^PD-L2^+^cells were sorted in RPMI 1640 + 10% FBS. The FACS gating strategy for TAMs is shown in **Supplementary Figure 1.**

### 2.13 Bone marrow-derived monocytes (BMDMs) isolation, differentiation, and polarization

BMDMs were prepared by isolating bone marrow cells from tibias and femurs of C57/B6J mice. In brief, 6-week-old C57BL/6J mice were sacrificed by cervical dislocation and sterilized with 75% ethanol. The skin at the root of hind legs was incised, and muscle tissue was removed from the bones with scissors. The bones were cut from both ends and flushed with DMEM medium using a 1 mL syringe. Bone marrow cells were cultured in DMEM containing 10% FBS, 1% penicillin-streptomycin mix (all from Gibco/Invitrogen Technologies, Waltham, Massachusetts, US) and 50 ng/mL macrophage colony stimulating factor (M-CSF, R&D systems, Minnesota, US) at 37°C in a 5% CO2 atmosphere for 7 d to obtain BMDMs. As shown in **Supplementary Figure 2**, to identify the purity of cells by flow cytometry, BMDMs were collected with a scraper, blocked with TruStain FcX (BioLegend, San Diego, California, US), and incubated with FITC anti-mouse CD45 (clone: I3/2.3), BV421 anti-mouse CD11b (clone: M1/70), and PE/Cy7 anti-mouse F4/80 (clone: BM8) for 15 min in the dark at 4°C. For M0, only DMEM-10% FBS was added. To derive M1 and M2 macrophages, BMDMs were treated with LPS (100 ng/mL, Sigma-Aldrich, Taufkirchen, USA) and recombinant IL-4 (20 ng/ml, PeproTech, Lpndon, UK) for 24 h, respectively. After polarization, the cells were collected for quantitative reverse transcription-polymerase chain reaction (qRT-PCR) analysis.

### 2.14 qRT-PCR analysis

M0, M1, and M2 macrophages, CD45^+^CD11b^+^F4/80^+^PD-L2^+^cells and CD45^+^CD11b^+^F4/80^+^PD-L2^-^cells were washed twice with PBS and total RNA was extracted using the TRIzol reagent (Invitrogen, Waltham, US). RNA quality and quantity were assessed using a Nanodrop ND-1000 Spectrometer (Thermo Scientific, Waltham, US). A total of 1 μg RNA of each sample was transcribed to complementary DNA (cDNA) using RevertAid First Strand cDNA Synthesis Kit (Thermo Scientific, Waltham, US) according to the manufacturer’s instructions. qRT-PCR reactions were performed using TB Green Premix Ex TaqTM II (Tli RNaseH Plus) (TaKaRa, Waltham, Japan) on a Quant-Applied Biosystems 7500 Fast Real-Time PCR system (Applied Biosystems, CA, USA). Each PCR reaction consisted of 2 μL of the cDNA as template, 0.8 μL (0.4 μM) of forward and reverse primers, 10.4 μL Mastermix, and 6 μL RNAnase-free water. Thermal cycle conditions consisted of denaturation at 95°C for 30 s, PCR at 95°C for 5 s and 60°C for 34 s. After 40 cycles, the reaction was completed with a final extension step at 95°C for 10 s and 55°C for 40 s. qRT-PCR primers sequences is listed in **Supplementary Table 2**. Each sample was run in triplicate and the levels of mRNA of a target were normalized to GAPDH. Fold induction was calculated using the 2^-△△Ct^ method.

### 2.15 Cell migration and invasion assays

For migration assays, MC38 colon cancer cell suspensions were evenly mixed with serum-free medium (200 μL, 2 × 10^4^ cells/well) and plated in the upper chamber of a 24-well Transwell plate (8 μm; Corning, New York, US). For invasion assays, 30 μL Matrigel matrix (pre-diluted 1:8 with serum-free medium; BD Biosciences, Franklin Lakes, New Jersey, US) was placed in the upper chamber of 24-Transwell plates (8 μm), and MC38 cells (200 μL, 2 × 10^4^ cells/well) were added after 2 h. Subsequently, TAMs were seeded in the lower chambers. After incubation for 24 h (migration) or 48 h (invasion) at 37°C and 5% CO2, tumor cell migration through the membrane was determined by fixing the cells for 10 min in 70% ethanol and staining with 1 × Giemsa (Beyotime Institute of Biotechnology, Shanghai, China) for 45 min. The inserts were washed with tap water after removing non-migrating cells with cotton swabs from the upper side of the filter and dried overnight. At least five random fields were selected for cell counting under a light microscope (Olympus Corp., Tokyo, Japan) at 200 × magnification.

### 2.16 Colony formation assay

For colony formation detection, MC38 cells (2 mL, 1000 cells/well) were uniformly seeded in the lower chamber of 6-well Transwell plate (0.4 μm; Corning, New York, US). TAMs (1 × 10^5^) were suspended in 2 mL of RPMI 1640 containing 10% FBS and added to the upper chamber. The cells were cultured in a humidified incubator at 37°C with 5% CO2 for 7 d. Tumor cells in the lower chamber were fixed with 70% ethanol for 10 min and stained with 1 × Giemsa for 45 min. The colonies were photographed using a high-resolution camera (Leica, MC 170HD) and counted.

### 2.17 Immunotherapy prediction analysis

Tumor Immune Syngeneic MOuse (TISMO) (http://tismo.cistrome.org), a database for investigating and visualizing gene expression, pathway enrichment, and immune cell infiltration levels in syngeneic mouse models across different immune checkpoint blockade (ICB) treatment and response groups (36), was used to identify the relationship between *pdcd1lg2* expression and ICB therapy response in mouse cohorts. Tumor Immune Dysfunction and Exclusion (TIDE) (http://tide.dfci.harvard.edu/) is a web platform can prioritize genes in an input gene set for mechanistic follow-up experiments, evaluate the accuracy of biomarkers on many ICB cohorts in comparison with other published biomarkers, and predict whether a patient responds to ICB therapy based on multiple biomarkers (37, 38). We used TIDE to verify the prediction performance of *pdcd1lg2* expression in human ICB therapy cohorts by applying the receiver operating characteristic (ROC) which measures the true-positive rates against the false-positive rates and the area under the ROC curve (AUC) which is an effective measure of accuracy. An AUC of 0.5 represents the performance of random predictor.

### 2.18 Drug sensitivity prediction

Gene Set Cancer Analysis (GSCALite) (http://bioinfo.life.hust.edu.cn/web/GSCALite/) is an online algorithm that integrates genomic and immunogenomic data of 33 cancer types from TCGA, drug responses from the Genomics of Drug Sensitivity in Cancer (GDSC) and the Cancer Therapeutics Response Portal (CTRP), and normal tissue data from GTEx (39). We used the GSCALite server to determine the correlation between *pdcd1lg2* mRNA expression and drug sensitivity [50% inhibitory concentration (IC50)] using the Pearson’s correlation analysis in the GDSC and CTRP databases.

### 2.19 Statistical analysis

All experiments were performed at least in triplicates. Data are presented as the mean ± standard deviation (SD). Statistical significance was determined using a two-tailed Student’s *t*-test for comparisons between two groups. All statistical analyses were performed using the SPSS software (version 17.0; SPSS Inc., US). Statistical significance was set at *p*<0.05.

## 3 Results

### 3.1 Multiple human cancers expressed *pdcd1lg2* mRNA and PD-L2 protein

**Figure 2:**
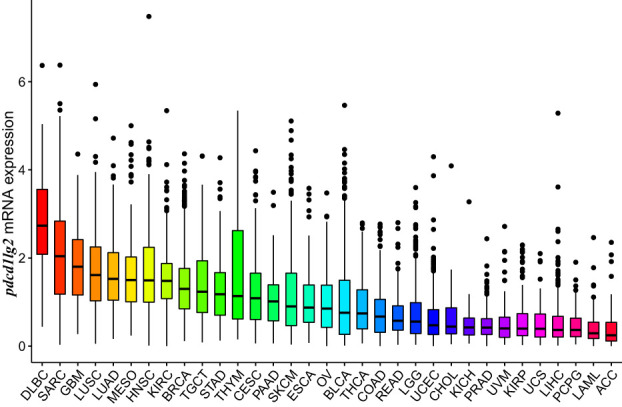
*Pdcd1lg2* expression in The Cancer Genome Atlas (TCGA) database.

The mRNA expression of *pdcd1lg2* was evaluated in pan-cancer patients of different cohorts according to the RNA-seq data of TCGA, the comprehensive program in cancer genomics that is jointly supported and managed by the National Cancer Institute and the National Human Genome Research Institute of the US National Institutes of Health, and ICGC database which is launched to coordinate large-scale cancer genome studies in tumors across the globe. As shown in **Figure 2** and **Supplementary Figure 3A**, all 33 types of common human cancer types in TCGA database and 12 types of human cancer queues in ICGC database expressed *pdcd1lg2*, though it exhibited inconsistent mRNA expression. Similarly, by analyzing the IHC images from HPA datasets to assess PD-L2 expression at the protein level in cancer tissues, we found that PD-L2 expression was unbalanced in cancers (**Supplementary Figure 3B** and **Supplementary Table 2**). It should be noted that PD-L2 could be expressed in both tumor and stromal cells.

### 3.2 *Pdcd1lg2* expression deteriorated outcomes of patients in multiple cancer types

We investigated the prognostic potential of PD-L2 by estimating the association between *pdcd1lg2* expression and survival of patients. According to Liu et al. (40), which provided recommendations of clinical outcome endpoint usage for 33 cancer types by analyzing the clinicopathologic annotations for over 11000 cancer patients in TCGA, OS and PFS could be derived relatively accurately and all four endpoints (OS, PFS, disease-free survival, and disease-specific survival) could be used in 13 of the 33 cancer types. Considering that OS has been historically considered as the “gold standard” and is the most objective endpoint used in clinical trials, OS would be used as the endpoint if it was applicable. As a result, OS was an appropriate endpoint for ACC, BLCA, CESC, CHOL, COAD, ESCA, GBM, HNSC, KIRC, KIRP, LAML, LIHC, LUAD, LUSC, MESO, OV, PAAD, SARC, SKCM, STAD, UCEC, UCS, and UVM. PFS was an appropriate endpoint for BRCA, LGG, PRAD, READ, TGCT, THCA, and THYM. In contrast, none of the four outcome endpoints could be recommended for use in the DLBC, KICH, and PCPG cases.

**Figure 3:**
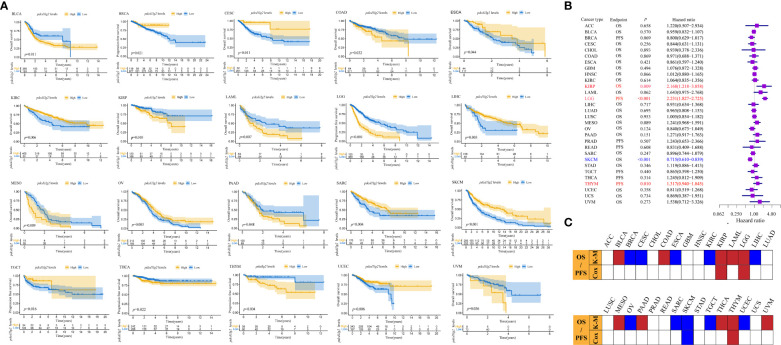
Prognostic values of *pdcd1lg2*. **(A)** The prognostic value of *pdcd1lg2* on overall survival (OS) or progression-free survival (PFS) displayed by the Kaplan-Meier method. **(B)** Cox regression analysis of *pdcd1lg2* on OS or PFS in pan-cancer described by the forest plot. **(C)** Based on the Kaplan-Meier models and Cox regression, summary of the correlation between *pdcd1lg2* expression and OS or PFS.

As shown in **Figures 3A, C** and **Supplementary Figure 4**, Kaplan-Meier survival curves indicated that high *pdcd1lg2* expression was significantly associated with the deteriorated outcomes in 10 cancer types by cutting the expression into dichotomous variables, including BLCA, COAD, KIRP, LAML, LGG, MESO, PAAD, THCA, THYM, and UVM. The results shown in forest plot (**Figures 3B, C**) demonstrated that *pdcd1lg2* expression upregulation was closely positively associated with poor prognosis in KIRP (HR=2.168 [95%CI, 1.218-3.858], *p*=0.009), LGG (HR=2.231 [95%CI, 1.827-2.725], *p<*0.001), and THYM (HR=1.317 [95%CI, 0.940-1.845], *p*=0.010) by taking the expression of *pdcd1lg2* as a continuous variable in Cox regression analysis.

### 3.3 *Pdcd1lg2* played an important role in cancer immune response

**Figure 4:**
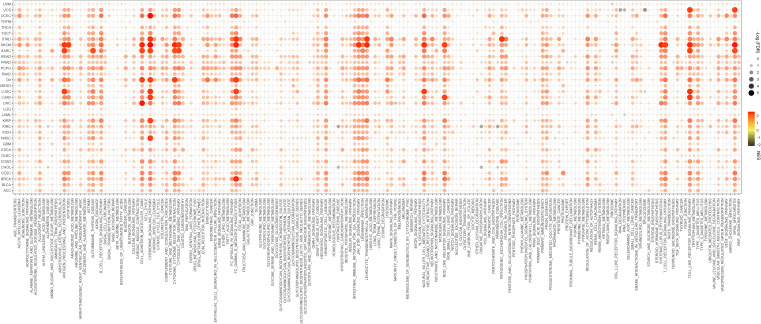
Signaling pathways associated with *pdcd1lg2* expression according to Kyoto Encyclopedia of genes and genomes (KEGG) analyzed by the Gene Set Enrichment Analysis (GSEA) in pan-cancer. FDR, false discovery rate; NES, normalized enrichment score.

To determine the biological processes associated with *pdcd1lg2* expression in cancers, we performed GSEA across 33 types of common human cancers in TCGA database. GSEA results of KEGG terms revealed that *pdcd1lg2* was involved in various pathways, including antigen processing and presentation, apoptosis, autoimmune thyroid disease, cell anhension molecules cams, chemokine signaling pathway, cytokine-cytokine receptor interaction, cytosolic DNA sensing pathway, FC gamma R mediated phagocytosis, JAK-STAT signaling pathway, leukocyte transendothelial migration, natural killer cell mediated cytotoxicity, NOD-like receptor signaling pathway, systemic lupus enythematosus, T cell receptor signaling pathway, TOLL-like receptor signaling pathway, and viral myocarditis signaling pathway (**Figure 4**).

Furthermore, the GSEA results for Reactome analysis also indicated that several immune functional gene sets were enriched in cancers, such as TCR signaling, TNFR2 non-canonical NF-κB pathway, Toll-like receptor cascades, signaling by interleukins, signaling by the B cell receptor BCR, FCERI mediated NF-κB activation, FCgamma receptor (FCGR) dependent phagocytosis, interleukin-12 family signaling, neutrophil degranulation, parasite infection, costimulation by the CD28 family, downstream signaling events of B cell receptor (BCR), activation of IRF3/IRF7 mediated by TBK1/IKK epsilon, and antigen processing cross presentation (**Supplementary Figure 5**).

**Figure 5:**
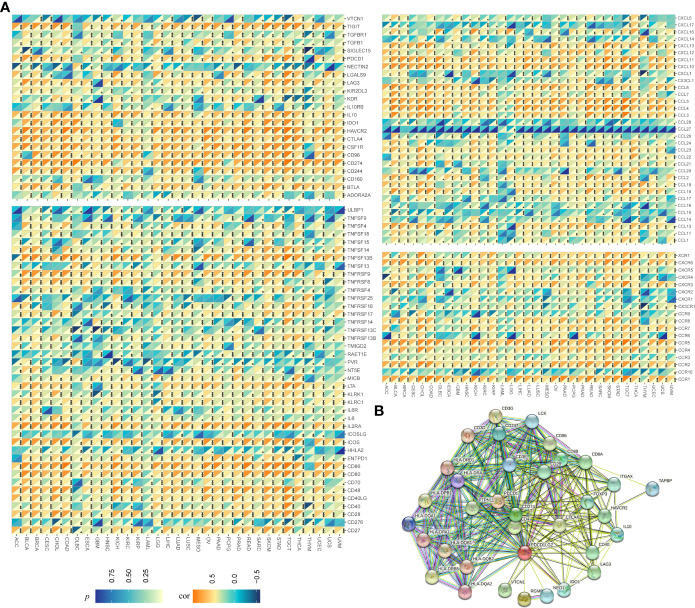
Relationship between *pdcd1lg2*/PD-L2 expression and immune-related genesin pan-cancer. **(A)** The Spearman correlation heatmap between *pdcd1lg2* expression levels and immune-related genes. **(B)** The protein-protein interaction (PPI) network presents the proteins interacting with PD-L2. *p<0.05, **p<0.01, ***p<0.001.

To confirm our suspicions, we further performed Spearman’s correlation analysis and constructed a PPI network. As shown in **Figure 5A**, the expressions of nearly all kinds of immune-related genes were significantly related to the expression of *pdcd1lg2* in pan-cancer. For example, the expressions of *TIGIT*, *IL-10*, *IDO1*, *HAVCR2*, *CTLA4*, *CSF1R*, *CD96*, *CD274*, and *CD244* which are immunosuppressive genes, were positively correlated with *pdcd1lg2* expression. Some immune activation genes including *ULBP1, TNFRSF25, TNFRSF14, TNFRSF13C, RAET1E, PVR, ICOSLG, HHLA2*, and *CD276* were negatively correlated with *pdcd1lg2* expression in some cancers. Moreover, the stimulation of directed migration of immune cells is the most prominent role of the large family of chemokines and their receptors. The results revealed that *pdcd1lg2* expression was positively related with the expressions of *CXCL13*, *CXCL11*, *CXCL10*, *CCL8*, *CCL5*, *CCL4*, and *CCL3* which are chemokines, and *CXCR6*, *CCR5*, *CCR4*, *CCR2* and *CCR1* which are chemokine receptor molecules, and was negatively associated with the expressions of other chemokines such as *CXCL5, CXCL17, CXCL1, CX3CL1, CCL28, CCL15* and *CCL14*, and other chemokine receptor such as *CXCR4, CXCR2, CXCR1, CX3CR1, CCR10,* and *CCR9* in some types of cancer. In addition, based on the information from the STRING database, PPI network revealed that PD-L2 was closely associated with CD3G, CD3E, CD3D, LCK, CD247, HLA-DRB1, HLA-DRB5, HLA-DQB2, HLA-DQB1, HLA-DPB1, HLA-DQA1, HAL-DQA2, HLA-DRA, HLA-DPA1, CD86, CD80, CD8A, CD28, ITGAX, TAPBP, HAVCR2, FOXP3, CD274, PDCD1, PTPN11, CD4, CTLA4, HAVCR2, IL-10, CD40, LAG3, IDO1, NEO1, RGMB, and VTCN1(**Figure 5B**).

### 3.4 PD-L2 mainly expressed in TAMs in colon cancer

To identify the immune aspects of PD-L2 in TME in pan-cancer, we calculated the correlation between *pdcd1lg2* levels and the immune scores (represent the infiltration of immune cells in tumor tissue) and stromal scores (capture the presence of stroma in tumor tissue) in 33 types of cancer based on the ESTIMATE algorithm. The results showed that *pdcd1lg2* expression was significantly and positively correlated with immune contexture and stromal contexture in almost all cancer types (**Supplementary Figure 6**).

**Figure 6:**
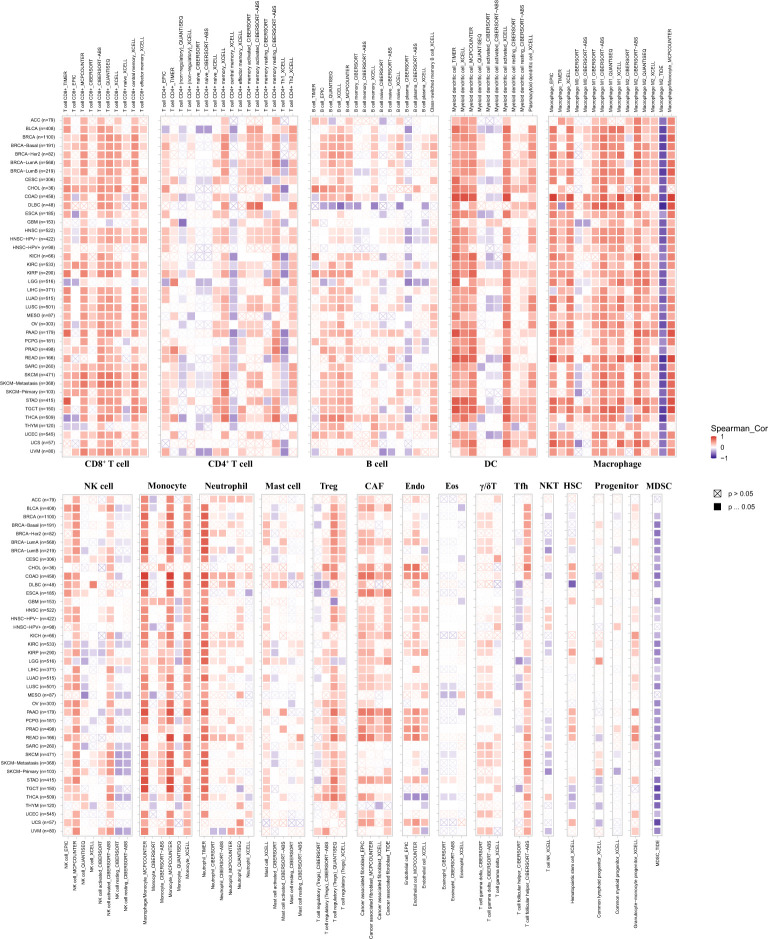
The correlation between the expression of *pdcd1lg2* and various immune cells infiltration levels in cancers. DC, dentritic cell; NK cell, natural killer cell; Treg, T cell regulatory; CAF, cancer-associated fibroblast; Endo, endothelial cell; Eos, eosinophil; γ/δ T cell, T cell gamma delta; Tfh, T cell follicular helper; NKT, nature killer T cell; HSC, hematopoietic stem cell; MDSC, myeloid-derived suppressor cell.

Then, we performed a pan-cancer analysis of relationships between *pdcd1lg2* expression and immune cell infiltration levels using TIMER 2.0. As shown in **Figure 6**, in general, *pdcd1lg2* expression was moderate positively and significantly associated with the amount of multiple infiltrating immune cells in various cancers, including CD8^+^T cell, DC, monocyte, Treg, CAF, and endothelial cell and weak positively and significantly related with the abundance of neutrophil, and γ/δT cell. However, the trends of correlations between *pdcd1lg2* expression and the abundance of CD4^+^T cell, B cell, NK cell, Tfh, and mast cell were different according to different algorithms. Additionally, a strong negative correlation was observed between the expression of *pdcd1lg2* and the infiltration of MDSC in all almost kinds of cancers. Notably, the abundance of macrophages was the most positively associated with *pdcd1lg2* expression in multiple types of cancer, especially in COAD (Rho=0.917 for macrophage EPIC; Rho=0.496 for macrophage TIMER; Rho=0.818 for macrophage XCELL; Rho=0.913 for macrophage/monocyte MCP-COUNTER).

**Figure 7:**
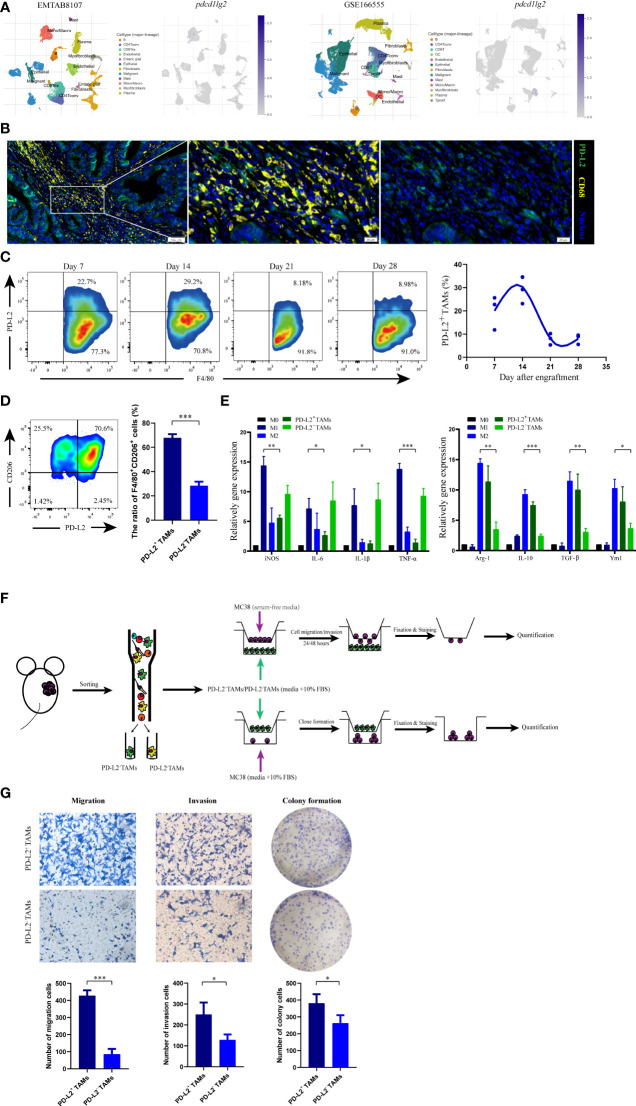
The phenotype and functions of PD-L2^+^ tumor associated macrophages (TAMs). **(A) **The localization of *pdcd1lg2 *expression analyzing in two scRNA-seq datasets. **(B)** The multiplex immunofluorescence images of PD-L2^+^TAMs in human colon cancer tissues. **(C)** Representative flow cytometry plots and analysis of the expression of PD-L2 on TAMs in MC38 tumors at different days after engraftment. **(D)** Flow cytometric analysis of the expression of CD206 on PD-L2^+^TAMs and PD-L2^-^TAMs. **(E)** M1 macrophage and M2 macrophage marker gene expressions in macrophages. **(F) **Schematic overview of the strategy for identification the functions of PD-L2^+^TAMs. **(G)** Tranwell assays and colony formation to detect the role of PD-L2^+^TAMs in the migration, invasion, and proliferation of MC38 cells. *p<0.05, **p<0.01, ***p<0.001.

To further confirm the localization of *pdcd1lg2 *expression in TAMs, we included and analyzed two scRNA-seq datasets, EMTAB8107 and GSE166555. As shown in **Figure 7A**, *pdcd1lg2* mainly located or bound to monocyte/macrophage in colorectal cancer.

Additionally, we verified the results by analyzing the human and murine specimens from the National Cancer Center. Multiplex immunofluorescence in colon tissue revealed a clear and abundant population of cells that expressed both PD-L2 and CD68, which has been widely recommended as a pan-macrophage marker in human, confirming PD-L2 expression on TAMs (**Figure 7B**). Furthermore, to assess PD-L2^+^TAMs in mice in an immunocompetent syngeneic setting, we used the colon cancer mouse cell line, MC38. Flow cytometry analysis of dissociated tumors showed that the PD-L2^+^TAMs population was not static (**Figure 7C** and **Supplementary Figure 7A**); it began to emerge approximately one week after engraftment (117.23 ± 23.26 mm^3^) and increased subsequently. Two weeks after engraftment into mice (298.03 ± 92.66 mm^3^), the highest percentage of macrophages in the tumor expressed surface PD-L2, which subsequently decreased. Therefore, PD-L2 expression was correlated with time after engraftment in mice and the tumor size. FACS was used to obtain TAMs from dissociated MC38 tumors 2 weeks after engraftment (**Supplementary Figure 7B**) for phenotype and function analysis.

### 3.5 PD-L2^+^TAMs exhibited protumor phenotype and function

In the immune contexture from tumor grafts, we used CD206, a well-established marker of M2 macrophages, to distinguish M2-like protumor TAMs. As shown in **Figure 7D**, flow cytometry showed that CD206^+^ subpopulations were more abundant in PD-L2^+^TAMs than in PD-L2^-^TAMs, which indicated that PD-L2 was mainly expressed in TAMs with protumor phenotype. Moreover, myeloid-derived macrophages are important innate immune cells that can be induced to differentiate into TAMs. To further demonstrate our hypothesis, we also carried out gene expression analysis of selected marker genes, consisting of M1 macrophage markers (iNOS, IL-6, IL-1β, and TNF-α) and M2 macrophage markers (Arg-1, IL-10, TGF-β, and Ym1), by employing BMDMs-derived macrophage phenotype as control. The results revealed that PD-L2^+^TAMs showed extremely low M1 polarization-related genes expression compared to LPS-driven M1 macrophages and similar M2 marker genes expression as IL-4-driven M2 macrophages (**Figure 7E**). Additionally, the relative mRNA expression levels of M1 phenotypic markers (IL-1β and TNF-α) in PD-L2^+^TAMs were downregulated by more than 5-fold as observed in PD-L2^-^TAMs, whereas the expression of M2-type markers (Arg-1, IL-10, and TGF-β) in PD-L2^+^TAMs were upregulated by more than 2-fold as observed in PD-L2^-^TAMs.

Next, we explored the role of PD-L2^+^TAMs in tumor development. In the study, the impact of PD-L2^+^TAMs on colon cancer cell migration, invasion, and proliferation was first analyzed (**Figure 7F**). In migration assays, the migration ability of MC38 cells was greater after incubation with PD-L2^+^TAMs than with PD-L2^-^TAMs [**Figure 7G** (left)]. Similar results were observed in invasion assays [**Figure 7G** (middle)]. To further verify whether PD-L2^+^TAMs directly induced the growth of colon tumor cells, we performed colony formation assays by co-culturing MC38 cells with PD-L2^+^TAMs/PD-L2^-^TAMs. As shown in **Figure 7G** (right), PD-L2^+^TAMs remarkably increased the number of new MC38 cell colonies compared with PD-L2^-^TAMs.

### 3.6 *Pdcd1lg2* could predict the response to cancer therapy and a series of targeted small-molecule drugs were identified

**Figure 8:**
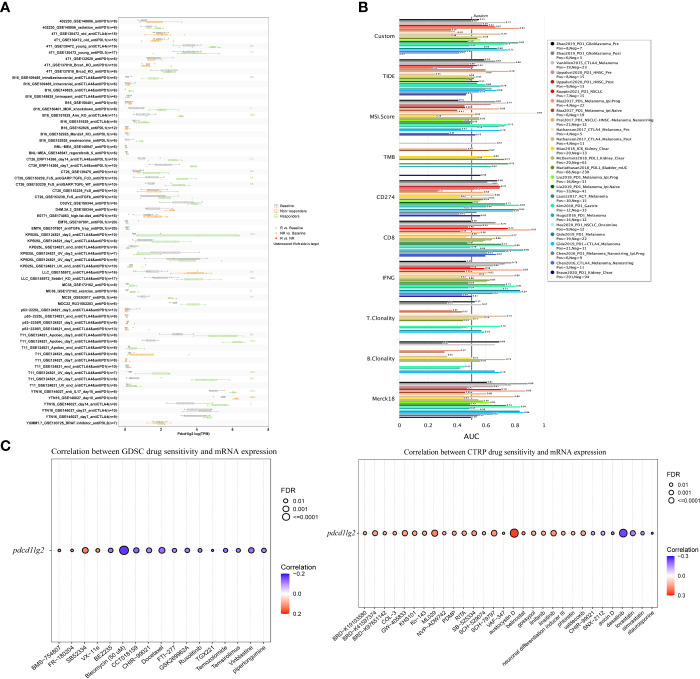
Immunotherapy response prediction, biomarker relevance and sensitive drug prediction of PD-L2. **(A)** Immunotherapy response of *pdcd1lg2* in murine immune checkpoint blockade (ICB) therapy cohorts analyzed by TISMO database. **(B)** Biomarker relevance of *pdcd1lg2* compared to standardized biomarkers with consistent evidence on cancer immune evasion in ICB therapy cohorts. The area under the receiver operating characteristic curve (AUC) is applied to evaluate the prediction performance of the biomarker on ICIs response status. **(C)** Predictive drugs based on the *pdcd1lg2* expression from the GDSC (left) and CTRP (right) databases.

As shown in **Figure 8A**, *pdcd1lg2* could significantly predict the ICB therapy response in 21 murine immunotherapy cohorts, wherein which the responders had elevated *pdcd1lg2* expression levels in 19 cohorts. Although *pdcd1lg2* expression was higher in non-responders in two cohorts, these two ICB cohorts did not have responders. We also verified the predictive value of *pdcd1lg2* in 25 human ICB therapy cohorts by comparing its predictive power with that of other standardized biomarkers. The results revealed that *pdcd1lg2* alone had an AUC of more than 0.5 in 15 ICIs therapy cohorts (**Figure 8B**), suggesting it to be a robust predictive biomarker, while microsatellite instability (MSI) score, tumor mutation burden (TMB), T. Clonality, and B. Clonality gave AUC values above 0.5 in 13, 8, 9, and 7 ICIs therapy cohorts, respectively. However, the predictive significance of *pdcd1lg2* was lower than that of CD274, CD8, and IFNG, which had AUC above 0.5 in 21, 18, and 17 ICIs therapy cohorts, respectively.

Finally, we predicted drug sensitivity based on *pdcd1lg2* expression according to data from the GDSC and CTRP datasets (**Figure 8C**). Based on the GDSC dataset, higher expression levels of *pdcd1lg2* were associated with increased sensitivity to BMS-754807 (insulin-like growth factor 1 receptor inhibitor), FR-180204 (extracellular signal-regulated kinase inhibitor), SB52334 (transforming growth factor-β receptor I inhibitor), and VX-11e (extracellular signal-regulated kinase inhibitor). The correlation between *pdcd1lg2* levels and drug sensitivity based on the CTRP dataset showed that austocystin D, ML029 (inhibitor of nuclear factor kappa B activation), SCH-79797 (proteinase-activated receptor 1 receptor antagonist), and linsitinib (inhibitor of both type 1 insulin-like growth factor receptor and the insulin receptor) were the top four drugs positively correlated with *pdcd1lg2* expression.

## 4 Discussion

Over the past few decades, research in cancer therapies focused on exploration of mechanisms of protective tumor immunity, which has provided several therapeutic strategies. Among these, immune checkpoint inhibitors can reverse the negative regulators of T cell function, which revolutionized cancer treatment and became the most dazzling star (5). Therapeutic antibodies for blocking PD-1 and PD-L1 have been developed and had early success in the clinic. However, since the clinical efficacy of current therapy strategies is limited and clinicians still have very limited tools to distinguish patients who will and will not respond to therapy, identification of new targets and predictive biomarkers are crucial to further improve patients’ survival.

Compared to PD-1 and PD-L1, PD-L2 has not received much attention and its role in modulating tumor progression is still being investigated. Our study not only uncovered the expression profile, prognostic value, and predictive potential of PD-L2 in a pan-cancer dataset for the first time, but also identified PD-L2^+^TAMs as immune effector cells with protumor function *in vivo* and *in vitro*.

By evaluating the association between *pdcd1lg2* and OS or PFS, we found that high *pdcd1lg2* expression was closely related to the deteriorated outcomes in BLCA, COAD, KIRP, LAML, LGG, MESO, PAAD, THCA, THYM, and UVM. These results are consistent with previous studies in bladder cancer (41), acute myeloid leukemia (42), and pancreatic ductal adenocarcinoma (43, 44). However, some studies revealed different conclusions. Qiao et al. found that high expression of PD-L2 was an independent predictor of poor OS in patients with HNSC. (45) and Takamori et al. revealed that PD-L2-positive lung adenocarcinoma patients had a significantly shorter OS (46). The discrepancies among the results may be due to the varying clinical features of the samples analyzed, such as the site and size of cancer, treatments, or different ethnic populations. Besides, PD-L2 is a dynamic marker that can be up- or down-regulated temporarily, verified by our *in vivo* experiments. Moreover, PD-L2 protein is expressed to varying degrees in stromal, endothelial, and tumor cells. Ariafar et al. (47) found that PD-L2 expression on immune cells, especially in draining lymph nodes was valuable for predicting prognosis and survival, while PD-L2 expression on tumor cells was not associated with prognosis. Therefore, it is necessary to explore the function and prognostic value of PD-L2 at a cellular level.

TME, which is a complex structure composed of tumor cells, nonmalignant cells, blood vessels, extracellular matrix, and other substances, plays a crucial role in stimulating cancer cells and increasing multidrug resistance, which result in cancer progression and metastasis (48, 49). Our results revealed an interesting phenomenon that *pdcd1lg2* expression was positively correlated with some immunosuppressive and immunostimulatory genes in the same group of patients, which further reflects the complexity of TME. In the TME, the PD-L1 and PD-L2 exhibit distinct patterns of expression. Apart from cancer cells, TAMs, DCs, activated T cells, activated B cells, and CAFs also express PD-L1. In contrast, PD-L2 expression is restricted (3, 50). In this study, the IHC information from the HPA dataset showed that PD-L2 was expressed in both tumor and stromal cells. There were three pieces of evidence that led us to focus on the immune cells expressed PD-L2. First, the results of GSEA demonstrated that *pdcd1lg2* played an important role in cancer immune response. Second, the expression of *pdcd1lg2* was related to that of most immune-related genes based on Spearman’s correlation and PPI analysis. Last, *pdcd1lg2* expression was significantly positively correlated to the immune score and stromal score in almost all kinds of cancers when analyzed using the ESTIMATE algorithm.

In 2002, Dunn et al. proposed the concept of cancer immunoediting as a result of three processes: elimination, equilibrium, and escape, which depended on different immune cells in TME (51). We performed a pan-cancer analysis using TIMER 2.0 and found that *pdcd1lg2* expression was significantly positively associated with multiple infiltrating immune cells in various cancers, especially macrophages in COAD. Macrophages in TME, referred to as TAMs, are a major TME component and the main regulator in response to various microenvironmental signals generated from tumor and stromal cells (52). An increasing number of studies have shown that the presence of TAMs correlates with tumor progression, poor clinical outcome, and the efficacy of therapeutics in various types of cancers, including colorectal cancer (CRC) (53–55). Since decades, engineering TAMs for cancer immunotherapy and drug delivery has been encouraging clinical applications (56). Given the crucial roles of TAMs, we validated whether TAMs expressed PD-L2 in the colon cancer microenvironment, and if so, the consequences that PD-L2 expression may have on tumor progression. Using multiplex immunofluorescence and flow cytometry, we found that PD-L2 was expressed in TAMs of both human clinical samples and mice syngeneic tumor models. Further analysis revealed that the PD-L2^+^ TAMs population was not static and changed over time. Moreover, the *in vitro* experimental evidence demonstrated the protumor functions of PD-L2^+^TAMs in colon cancer. CRC exhibits an immunosuppressive TME and the benefits of current immunotherapies in CRC are limited to a few groups of patients with microsatellite instability-high tumors (57, 58). New therapeutic approaches that do not only benefit a selected group of CRC patients are highly crucial. In additional to the direct promotion of tumor cell growth, migration, and invasion of PD-L2^+^TAMs identified in our studies, the previous studies also revealed that PD-L1 and PD-L2 had similar protein structures and the affinity of PD-L2 to PD-1 was two to six-fold higher than that of PD-L1. This is because PD-L1 binding to PD-1 requires complex conformational changes in the ligand, whereas PD-L2 directly binds to PD-1 (59, 60), which demonstrates that PD-L2 would outcompete PD-L1 in binding to PD-1 and could be a means by which cancer cells evade the immune system. Therefore, although more evidences of the therapeutic effect of PD-L2 in TAMs are required, our study suggested that PD-L2 signaling in TAMs showed potential as a novel therapeutic target.

Apart from TAMs, Treg cells, known as the immunosuppressive class of CD4^+^T cells suppress anti-cancer immunity (61), and CAFs, which promote tumor growth, angiogenesis, invasion and metastasis and remodel extracellular matrix (62), were also positively associated with *pdcd1lg2* expression in this study. Moreover, Tanegashima et al. (63) showed that PD-L2 expression in tumor cells also played an important role in evading antitumor immunity. These results can be the primary domain for future studies.

However, our results also revealed that *pdcd1lg2* expression was moderate positively correlated with CD8^+^T cells, which are killer cells in the TME, and negatively correlated with MDSCs, which exert immunosuppressive effects by suppressing T cell activity (64). This finding may partially explain the protective role of PD-L2 in some tumor types such as SKCM.

A highly positive correlation between *CD274* (PD-L1-encoding gene) and *pdcd1lg2* expression in almost all cancers was observed in our results, which might suggest that targeting PD-L1 will not show apparent benefits since PD-L2 is still functional and plays a redundant role. In these cases, combinational therapies against PD-L1 and PD-L2 may further optimize the efficacy. In other words, PD-L2 blockade is necessary for controlling PD-L2-expressing tumors (45, 65). We also explored several targeted small-molecule drugs with promising therapeutic effects, providing a theoretical basis to develop drugs targeting PD-L2.

In addition to studying new immune checkpoints, the prediction of the cancer immunotherapy effect is another requirement for clinical application. Therefore, we also verified the promising predictive value of PD-L2 in murine and human immunotherapy cohorts and found that responders had elevated *pdcd1lg2* expression levels in 19 murine immunotherapy cohorts. In 25 human ICB therapy cohorts, *pdcd1lg2* exhibited a higher predictive value than MSI score, TMB, T. Clonality, and B. Clonality. Similarly, other studies also showed that expression of PD-L2 had a predictive value for response to pembrolizumab (66, 67), and MPDL3280A treatment (68). Notably, a predictive value is primarily observed for PD-L2 expression on both immune and tumor cells, thus further studies should also focus on the predictive capacity of PD-L2 expression on immune infiltrating cells, or tumor cells alone. Interestingly, some studies revealed that PD-L2 expression was also correlated with the efficacy of Bacillus-Calmette Guerin vesicle in bladder cancer patients (69) and rituximab, cyclophosphamide, doxorubicin, vincristine, and prednisone chemotherapy in DLBC patients (70).

By exploring and incorporating information from several databases and validating them in experiments, there are two implications can be directly applied to further studies. First, the protumor functions of TAMs are executed by expressing cytokines, chemokines, enzymes, and cell surface receptors to activate Treg cells or suppress other effector cells (54, 71). We have verified that PD-L2^+^ TAMs promote migration, invasion, and proliferation of cancer cells in colon cancer, and further in-depth mechanistic analysis *in vitro* or *in vivo* is required to validate our results. Second, this study has confirmed the potential ability of PD-L2 in predicting ICIs therapy response. Further investigations need to be carried out clinically and mechanistically in individual cancer types.

In addition, surface plasmon resonance analysis revealed that the affinity of PD-L2 for PD-1 was 2-fold to 6-fold higher than PD-L1 (59, 60). Lázár-Molnár E and colleagues even showed that PD-L2 had a 30-fold higher affinity for PD-1 than PD-L1 (72). This high-affinity binding can be the attractive target for the drug development with small compounds (73). Moreover, there are three isoforms of PD-L2 identified and both isoforms II and III can be interact with PD-1, while type I form supposedly loses the capacity to bind PD-1 (74, 75). Xiao Y et al. (76) found repulsive guidance molecule b, a co-receptor for bone morphogenetic protein, could also interact with PD-L2 and this interaction inhibited the invasion and metastasis of bladder and breast cancer (77, 78). The function of PD-L2 is complex, and there is still a long way to go to study it.

In conclusion, we comprehensively assessed the expression profiles, prognostic and predictive value and functions of PD-L2 in pan-cancer. We also investigated the phenotype and protumor functions of PD-L2^+^TAMs. Therapies targeting PD-L2 in the TME, especially TAMs, are promising for improving and prolonging the survival of cancer patients.

## Data availability statement

The original contributions presented in the study are included in the article/**Supplementary Material.** Further inquiries can be directed to the corresponding author.

## Ethics statement

The studies involving human participants were reviewed and approved by the medical ethics committee of the National Cancer Center. Informed consent was obtained from all patients enrolled in this study. The animal study was reviewed and approved by the ethics committee of the Chinese Academy of Medical Sciences, National Cancer Center.

## Author contributions

Writing-Original Draft, Methodology, and Visualization: JFL. Validation and Conceptualization: JFL, ZJ and JHY. Investigation: JFL, MZ and JHY. Methodology: HCL, XG, YFY and YMM. Project Administration: ZL, ZJ and HYW. Supervision, Project Administration and Funding Acquisition: XSW. All authors contributed to the article and approved the submitted version.
