# Supplementary material for: Pan-cancer analysis identifies PD-L2 as a tumor promotor in the tumor microenvironment
Source: Front Immunol. 2023 Mar 16;14:1093716. doi: 10.3389/fimmu.2023.1093716 (PMC10060638; doi:10.3389/fimmu.2023.1093716)
Supplement: Supplementary file 1 [file DataSheet_1.docx]

Supplementary Material

# Supplementary Figures and Tables

## Supplementary Figures


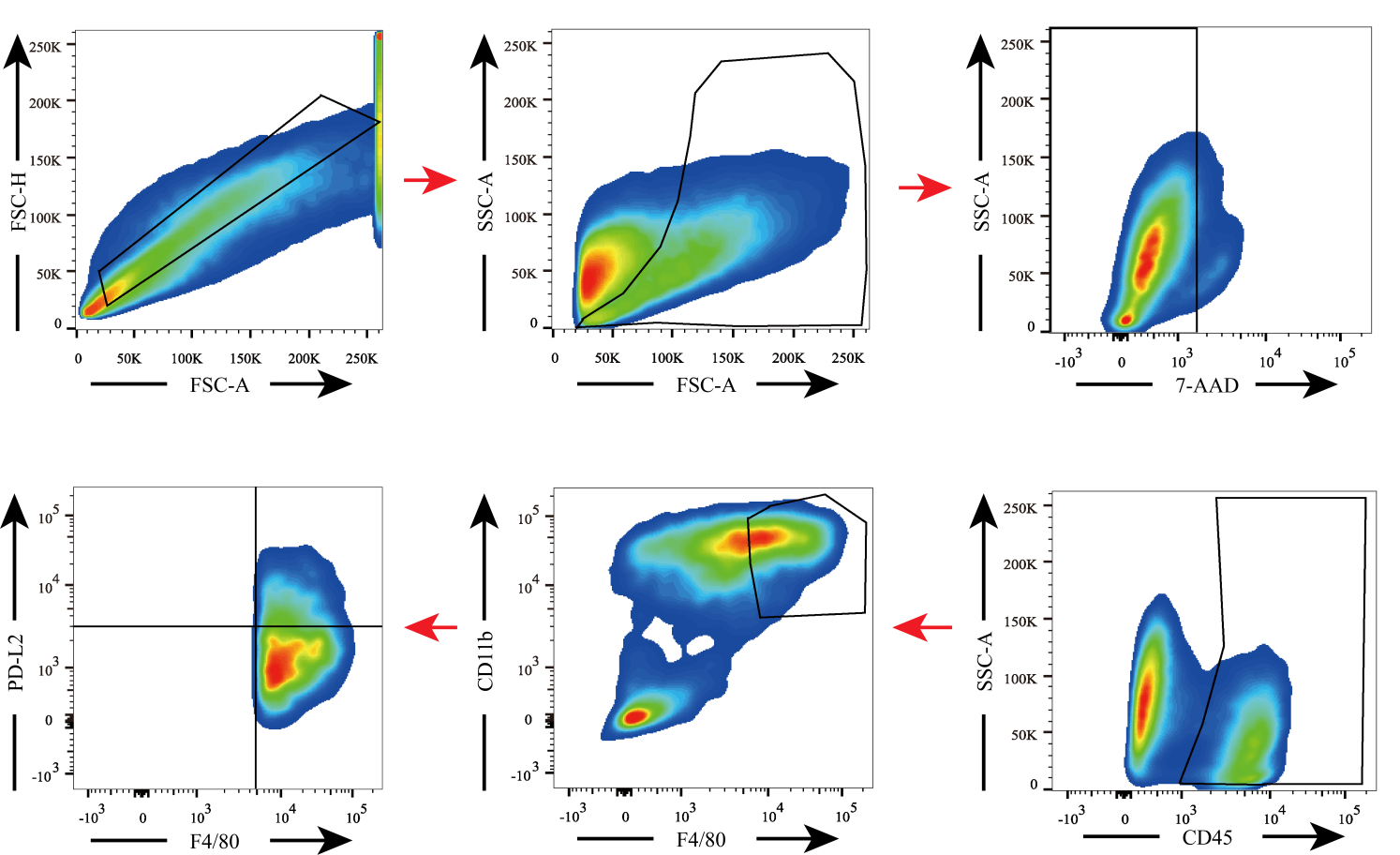


**Supplementary Figure 1.** Fluorescence-activated cell sorting gating strategy for PD-L2^+^ tumor associated macrophages (TAMs). Debris and doublets were removed, then PD-L2^+^TAMs were assessed as CD45^+^ CD11b^+^ F4/80^+^. All gates were determined on the basis of FMOs.


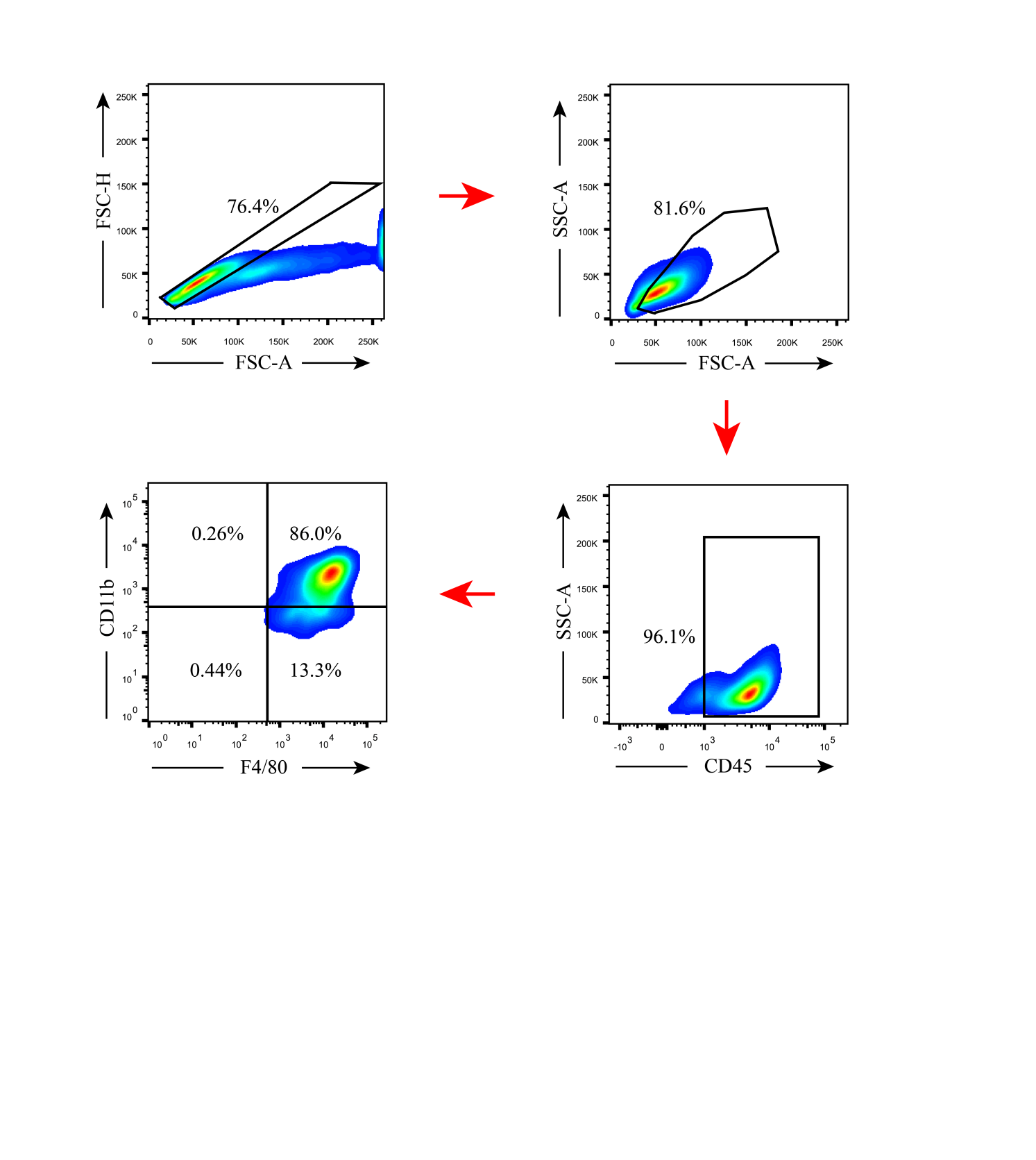


**Supplementary Figure 2.** Flow cytometric analysis of CD11b and F4/80 expression in Bone Marrow-Derived Monocytes (BMDMs)

**
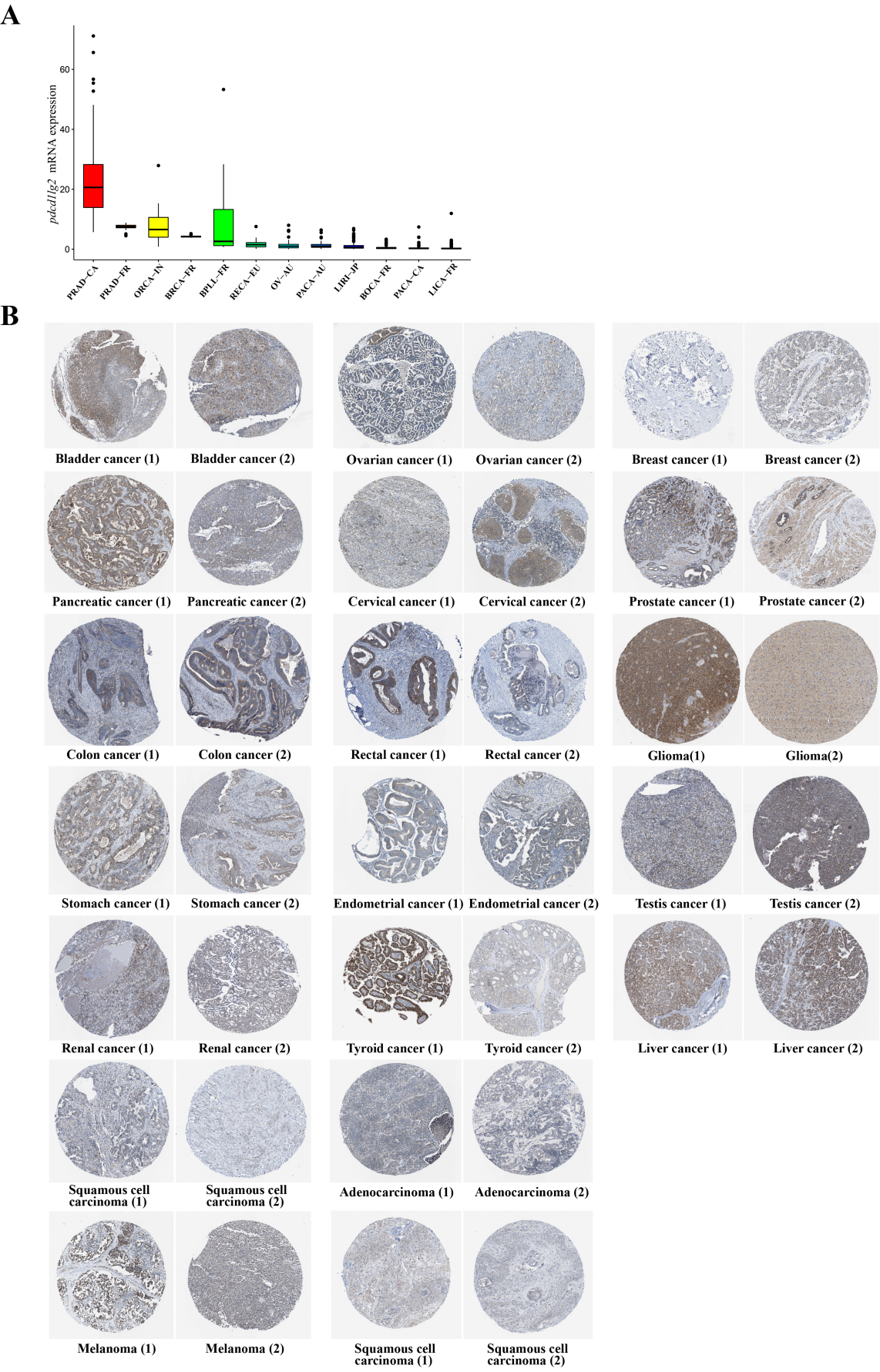
**

**Supplementary Figure 3.** Expression profile of *pdcd1lg2* mRNA and PD-L2 protein. **(A)** *Pdcd1lg2* expression in International Cancer Genome Consortium (ICGC) database. **(B)** The immunohistochemistry images of PD-L2 in cancer tissues.


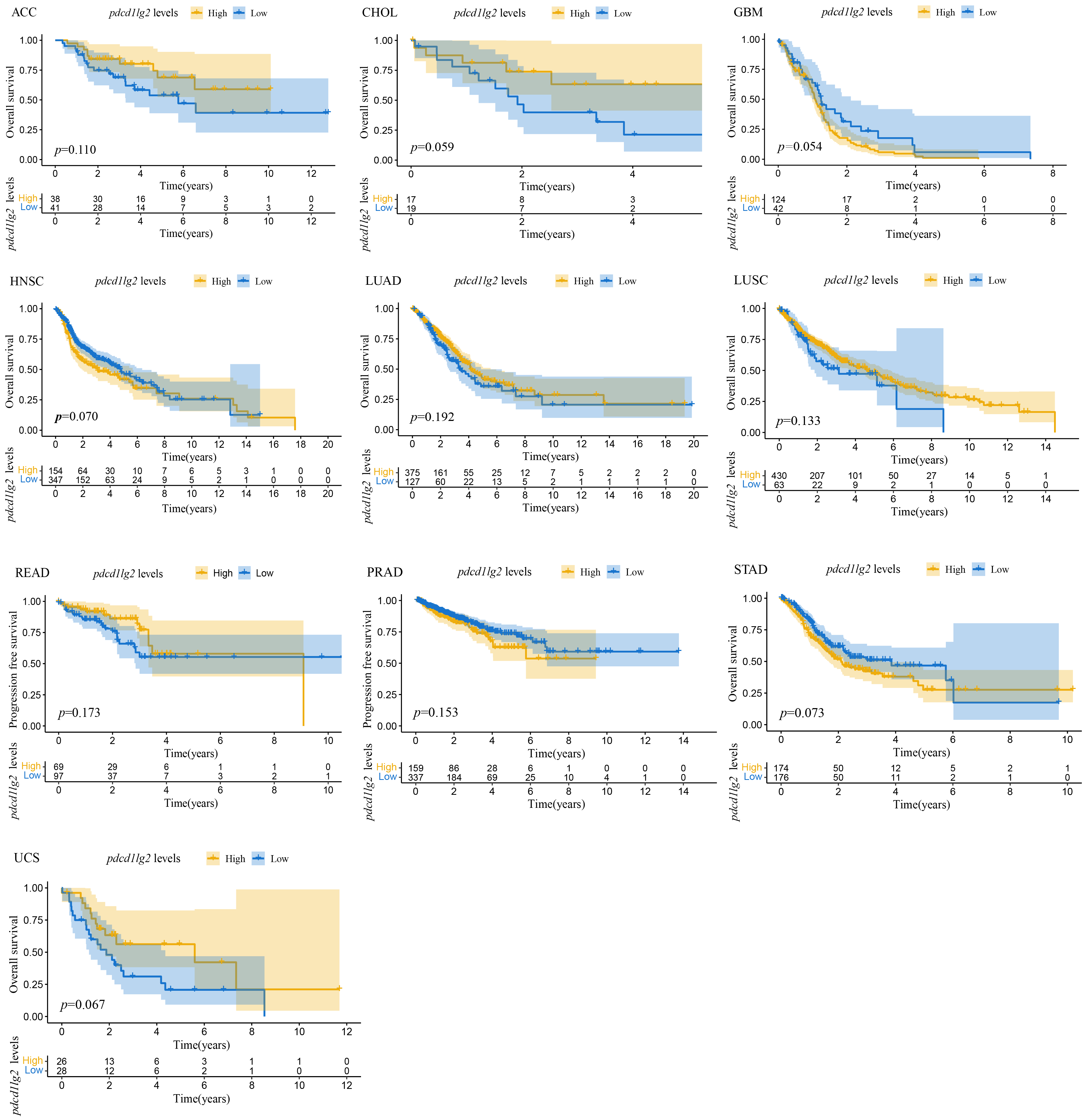


**Supplementary Figure 4.** Kaplan-Meier analysis to visualize the overall survival (OS) /progression free survival (PFS) curves.

**
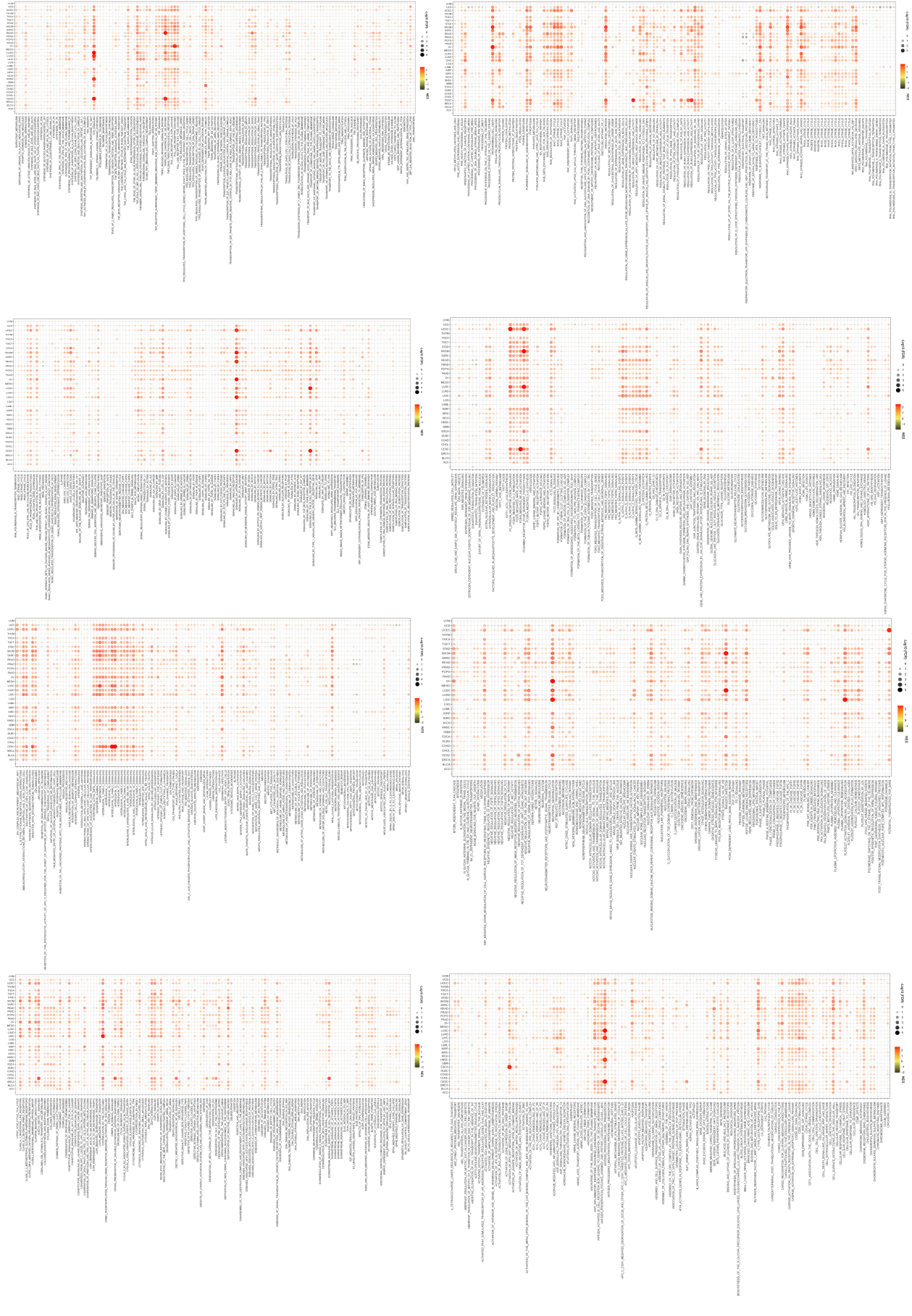
**

**Supplementary Figure 5.** Enrichment plots for *pdcd1lg2* obtained from Gene Set Enrichment Analysis (GSEA) according to Reactome analysis. Abbreviation: FDR, false discovery rate; NES, normalized enrichment score.


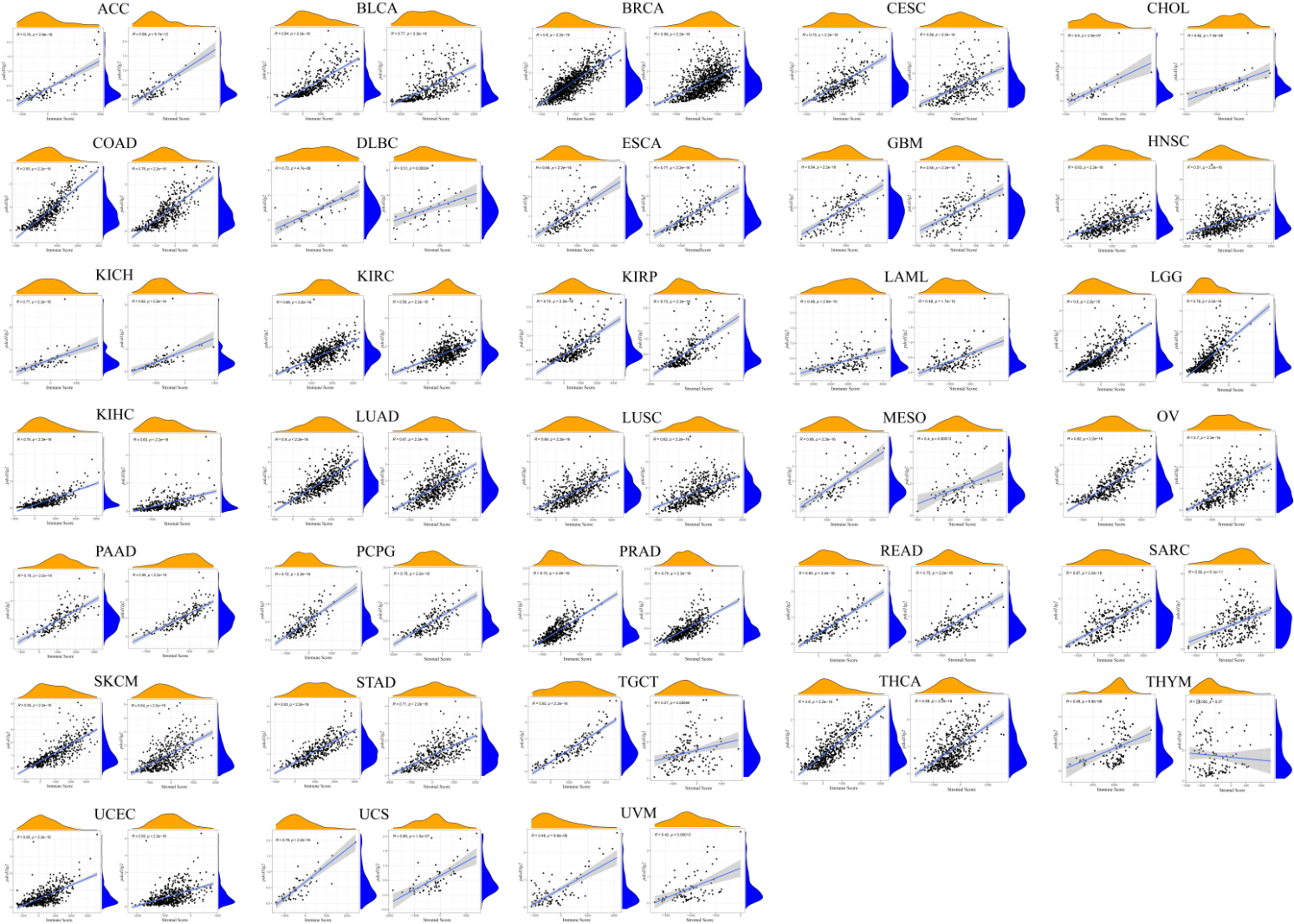


**Supplementary Figure 6.** Relationship between *pdcd1lg2* expression and the immune scores and stromal scores.

**
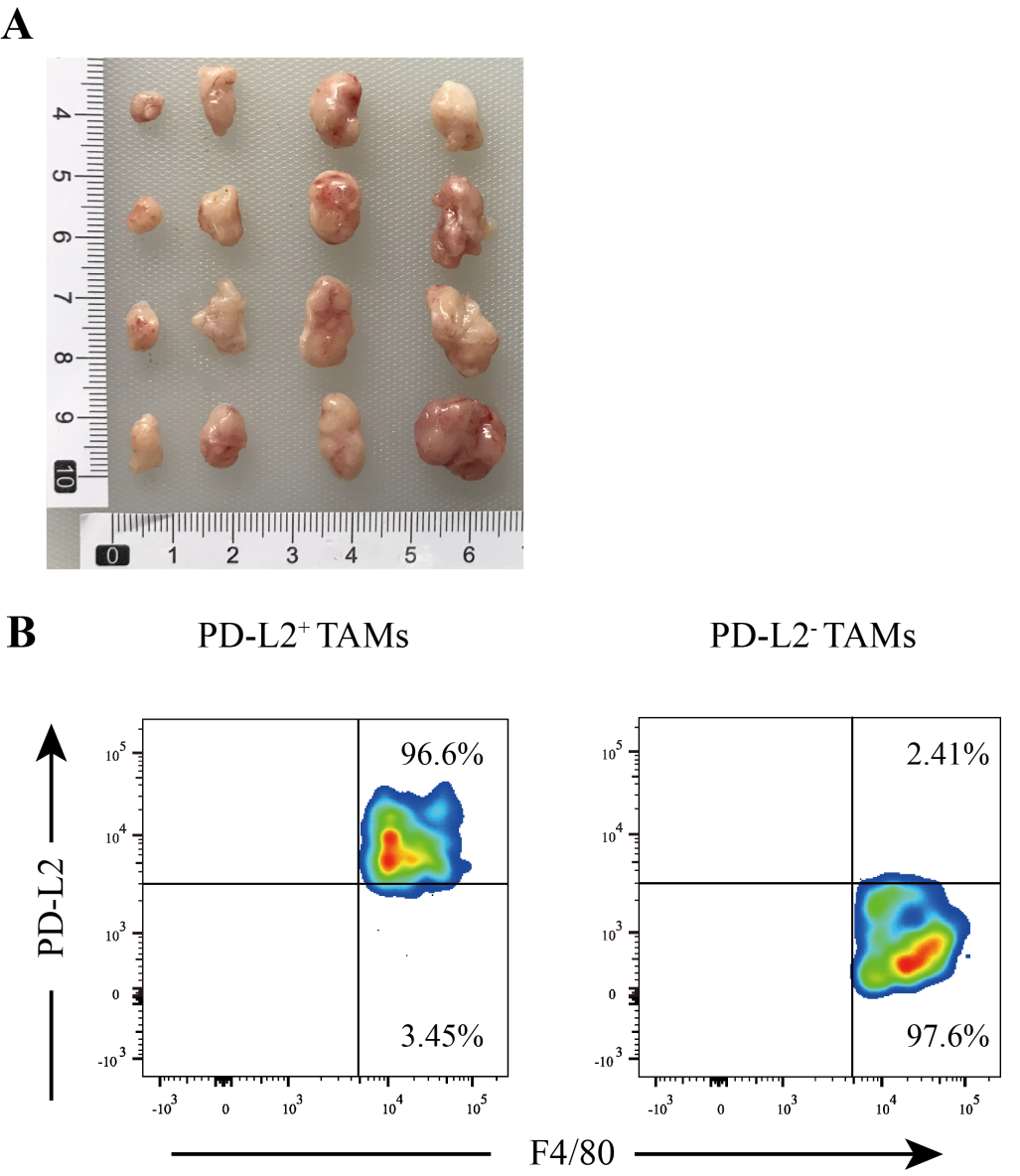
**

**Supplementary Figure 7.** Tumor information of Xenograft tumors **(A)** and the representative flow cytometry plots of fluorescence-activated cell sorting (FACS)-sorted PD-L2^+^tumor associated macrophages (TAMs) and PD-L2^-^TAMs **(B)**.

## Supplementary Tables

**Supplemental Table 1 Abbreviations for cancer**

| **Type of cancer** | **Abbreviation** |
| --- | --- |
| Adrenocortical carcinoma | ACC |
| Bladder urothelial carcinoma | BLCA |
| Breast invasive carcinoma | BRCA |
| Cervical squamous cell carcinoma and endocervical adenocarcinoma | CESC |
| Cholangiocarcinoma | CHOL |
| Colon adenocarcinoma | COAD |
| Lymphoid neoplasm diffuse large B-cell lymphoma | DLBC |
| Esophageal carcinoma | ESCA |
| Glioblastoma multiforme | GBM |
| Head and neck squamous cell carcinoma | HNSC |
| Kidney chromophobe | KICH |
| Kidney renal clear cell carcinoma | KIRC |
| Kidney renal papillary cell carcinoma | KIRP |
| Acute myeloid leukemia | LAML |
| Brain lower grade glioma | LGG |
| Liver hepatocellular carcinoma | LIHC |
| Lung adenocarcinoma | LUAD |
| Lung squamous cell carcinoma | LUSC |
| Mesothelioma | MESO |
| Ovarian serous cystadenocarcinoma | OV |
| Pancreatic adenocarcinoma | PAAD |
| Pheochromocytoma and paraganglioma | PCPG |
| Rectum adenocarcinoma | READ |
| Sarcoma | SARC |
| Skin cutaneous melanoma | SKCM |
| Stomach adenocarcinoma | STAD |
| Testicular germ cell tumors | TGCT |
| Thyroid carcinoma | THCA |
| Thymoma | THYM |
| Uterine corpus endometrial carcinoma | UCEC |
| Uterine carcinosarcoma | UCS |
| Uveal melanoma | UVM |
| Soft tissue cancer-Ewing sarcoma-France | BOCA-FR |
| B-cell prolymphocytic leukemia-France | BPLL-FR |
| Breast cancer-France | BRCA-FR |
| Liver cancer-France | LICA-FR |
| Liver cancer-RIKEN, Japan | LIRI-JP |
| Oral cancer-India | ORCA-IN |
| Ovarian cancer-Australia | OV-AU |
| Pancreatic cancer-Australia | PACA-AU |
| Pancreatic cancer-Canada | PACA-CA |
| Prostate adenocarcinoma-Canada | PRAD-CA |
| Prostate cancer-adenocarcinoma-France | PRAD-FR |
| Renal cell cancer-European Union/France | RECA-EU |

**Supplementary Table 2 The primers for qRT-PCR**

| **Target gene** | **Forward primer** | **Reverse primer** |
| --- | --- | --- |
| GAPDH | 5’-TACAGCAACAGGGTGGTGGAC-3’ | 5’-TGGGATAGGGCCTCTCTTGCT-3’ |
| iNOS | 5’-CAAGCACCTTGGAAGAGGAG-3’ | 5’-AAGGCCAAACACAGCATACC-3’ |
| IL-6 | 5’-CTGGAGCCCACCAAGAACGA-3’ | 5’-GCCTCCGACTTGTGAAGTGGT-3’ |
| IL-1β | 5’-AGCTTCCTTGTGCAAGTGTCT-3’ | 5’-GACAGCCCAGGTCAAAGGTT-3’ |
| TNF-α | 5’-CCAGGCAGGTTCTGTCCCTT-3’ | 5’-ATAGGCACCGCCTGGAGTTC-3’ |
| Arg-1 | 5’-AAGAATGGAAGAGTCAGTGTGG-3’ | 5’-GGGAGTGTTGATGTCAGTGTG-3’ |
| IL-10 | 5’-CTTACTGACTGGCATGAGGATCA-3’ | 5’-GCAGCTCTAGGAGCATGTGG-3’ |
| TGF-β | 5’-CCACCTGCAAGACCATCGAC-3’ | 5’-CTGGCGAGCCTTAGTTTGGAC-3’ |
| Ym-1 | 5’-CAGGTCTGGCAATTCTTCTGAA-3’ | 5’-GTCTTGCTCATGTGTGTAAGTGA-3’ |

**Supplemental Table 3 The results of immunohistochemistry (IHC) analysis of PD-L2 expression levels in tumor tissues from Human Protein Atlas (HPA) database**

| **Type of tissue** | **Cells/Histology** | **Antibody staining** | **Intensity** | **Quantity** |
| --- | --- | --- | --- | --- |
| Lung cancer (1) | Squamous cell carcinoma | Medium | Moderate | >75% |
| Lung cancer (2) | Squamous cell carcinoma | Low | Weak | >75% |
| Lung cancer (3) | Adenocarcinoma | Medium | Moderate | >75% |
| Lung cancer (4) | Adenocarcinoma | Low | Moderate | <25% |
| Breast cancer (1) | Lobular carcinoma | Low | Weak | >75% |
| Breast cancer (2) | Duct carcinoma | Medium | Moderate | >75% |
| Cervical cancer (1) | Squamous cell carcinoma | Low | Weak | 75%-25% |
| Cervical cancer (2) | Squamous cell carcinoma | Medium | Moderate | >75% |
| Colon cancer (1) | adenocarcinoma | Medium | Moderate | >75% |
| Colon cancer (2) | adenocarcinoma | Medium | Moderate | >75% |
| Glioma (1) | Glioma | High | Strong | >75% |
| Glioma (2) | Glioma | Medium | Moderate | 75%-25% |
| Rectal cancer (1) | adenocarcinoma | High | Strong | >75% |
| Rectal cancer (2) | adenocarcinoma | Medium | Moderate | 75%-25% |
| Endometrium cancer (1) | adenocarcinoma | Medium | moderate | >75% |
| Endometrium cancer (2) | Adenocarcinoma | Medium | Moderate | >75% |
| Liver cancer (1) | Hepatocellular | High | Strong | >75% |
| Liver cancer (2) | Hepatocellular | Medium | Moderate | >75% |
| Melanoma (1) | Melanoma | High | Strong | >75% |
| Melanoma (2) | Melanoma | Medium | Moderate | >75% |
| Skin cancer (1) | Squamous cell carcinoma | Medium | Moderate | 75%-25% |
| Skin cancer (2) | Squamous cell carcinoma | Low | Weak | 75%-25% |
| Ovarian cancer (1) | Cystadenocarcinoma, serous | Medium | Moderate | >75% |
| Ovarian cancer (2) | Cystadenocarcinoma, serous | Medium | Moderate | >75% |
| Pancreatic cancer (1) | Adenocarcinoma | High | Strong | >75% |
| Pancreatic cancer (2) | Adenocarcinoma | Medium | Moderate | >75% |
| Prostate cancer (1) | Adenocarcinoma | Medium | Moderate | >75% |
| Prostate cancer (2) | Adenocarcinoma | Medium | Moderate | >75% |
| Renal cancer (1) | Adenocarcinoma | High | Strong | 75%-25% |
| Renal cancer (2) | Adenocarcinoma | Medium | Moderate | >75% |
| Stomach cancer (1) | Adenocarcinoma | High | Strong | >75% |
| Stomach cancer (2) | Adenocarcinoma | Medium | Moderate | >75% |
| Testis cancer (1) | Seminoma | High | Strong | 75%-25% |
| Testis cancer (2) | Seminoma | Medium | Moderate | >75% |
| Thyroid cancer (1) | Papillary adenocarcinoma | High | Strong | >75% |
| Thyroid cancer (2) | Pollicular adenoma carcinoma | Not detected | Negative | None |
| Bladder cancer (1) | Urothelial carcinoma | High | Strong | 75%-25% |
| Bladder cancer (2) | Urothelial carcinoma | Medium | Moderate | >75% |
